# A Hybrid Dynamic Encryption Scheme for Multi-Factor Verification: A Novel Paradigm for Remote Authentication

**DOI:** 10.3390/s20154212

**Published:** 2020-07-29

**Authors:** Muath Obaidat, Joseph Brown, Suhaib Obeidat, Majdi Rawashdeh

**Affiliations:** 1 Center for Cybercrime Studies, City University of New York, New York, NY 10019, USA; joseph.brown1@jjay.cuny.edu; 2 Computer Science Department, Bloomfield College, Bloomfield, NJ 07003, USA; suhaib_obeidat@bloomfield.edu; 3 Department of Business Information Technology, PSUT, Amman 11941, Jordan; m.rawashdeh@psut.edu.jo

**Keywords:** authentication, cryptography, encryption, sessions, password, security, hashing, privacy, hybrid, communications, cyber-attack, MiTM, replay-attack, brute-force

## Abstract

A significant percentage of security research that is conducted suffers from common issues that prevent wide-scale adoption. Common snags of such proposed methods tend to include (i) introduction of additional nodes within the communication architecture, breaking the simplicity of the typical client–server model, or fundamental restructuring of the Internet ecosystem; (ii) significant inflation of responsibilities or duties for the user and/or server operator; and (iii) adding increased risks surrounding sensitive data during the authentication process. Many schemes seek to prevent brute-forcing attacks; they often ignore either partially or holistically the dangers of other cyber-attacks such as MiTM or replay attacks. Therefore, there is no incentive to implement such proposals, and it has become the norm instead to inflate current username/password authentication systems. These have remained standard within client–server authentication paradigms, despite insecurities stemming from poor user and server operator practices, and vulnerabilities to interception and masquerades. Besides these vulnerabilities, systems which revolve around secure authentication typically present exploits of two categories; either pitfalls which allow MiTM or replay attacks due to transmitting data for authentication constantly, or the storage of sensitive information leading to highly specific methods of data storage or facilitation, increasing chances of human error. This paper proposes a more secure method of authentication that retains the current structure of accepted paradigms, but minimizes vulnerabilities which result from the process, and does not inflate responsibilities for users or server operators. The proposed scheme uses a hybrid, layered encryption technique alongside a two-part verification process, and provides dynamic protection against interception-based cyber-attacks such as replay or MiTM attacks, without creating additional vulnerabilities for other attacks such as bruteforcing. Results show the proposed mechanism outperforms not only standardized methods, but also other schemes in terms of deployability, exploit resilience, and speed.

## 1. Introduction

Authentication is one of the most important structural components within wider, secure cyber-systems. However, while many aspects of how we maintain and secure systems have continued to evolve, accepted norms of authentication have not undergone significant changes in a similar parallel. Specifically, the usage of username/password style authentication has remained a controversial methodology despite its widespread usage. Not only has it been widely accepted that this methodology is increasingly flawed and insecure, but the continued advance of technology, and thus processing speeds, has continued to further this insecurity.

As individual factors within our lives continue to be increasingly digitized, especially sensitive information thanks to the rise of financial tech apps for example, the reliance on outdated, vulnerable methodologies is an increasingly dire threat. Security firm WhiteHat Security released a report in 2018 which estimated that at least 85% of mobile applications, regardless of categorization, possessed at least one violation of Open Web Application Security Project (OWASP) guidelines [1]. Insecure authentication, insufficient cryptography, and insecure authorization are among the most common issues found with many of these applications [2]. Moreover, with the explosive proliferation of the Internet of Things (IoT) devices in our daily lives, this has created an Internet of Vulnerabilities (IoV) [3,4,5]. Some studies have proposed outsourcing an authentication mechanism that requires IoT RFID-based devices to use a server on the edge of the network to complete the verification and the authentication process [5], which provides an increasingly secure communications by measuring four different security factors. Our proposed scheme can be extended with minor modification to accomplish this task without increasing the complexity of the IoT ecosystem.

Password processes with additional user-responsible factors such as CAPTCHA or two-factor authentication (2FA) over email or phone have been introduced. This inflation of user responsibilities has, in part, acted as a detriment to brute-forcing, but vulnerabilities to aforementioned attacks are still widespread [6,7,8].

This study proposes a methodology which attempts to set forth a new paradigm for authentication without dipping into any of the common pitfalls noted above. This method uses a hybrid, layered encryption scheme for dynamic, two-way, multi-factor verification. Based on comparisons to surveyed prior work in this field, this proposal introduces a new authentication process which aims to achieve the following: (i) it does not inflate any user responsibilities and/or add unnecessary secondary or tertiary complexities for the user; (ii) it does not require any restructuring of commonly-used remote or local Internet ecosystems, or the functional introduction of third party processes/software; (iii) it does not sacrifice processing speed; (iv) it provides theoretical flexibility for integration with proposals set forth in other studies for combating other forms of cyber-attacks; (v) on average outperforms other adjacent proposals for resilience to interception or impersonated-based cyber-attacks, speed, and user-responsibilities; (vi) in achieving the aforementioned goals, does not create further weaknesses to other types of attacks, such as brute forcing; and (vii) provides previously mentioned traits in the simplest means of implementation with regard to the highest relative security by comparison.

In comparison to other past studies on authentication, the proposal outlined herein provides a concept-tested scheme that is focused on countering attacks which result from impersonation and interception. The outlined scheme does this through a client–server relationship in which both sides verify each other to create an environment in which tokens can be exchanged and compared against local data to verify the counterpart without explicit, sensitive data being exchanged. Once that has been done, if there is no other means of starting a session, the paper then shows how the authentication scheme can be extended to provide a secure session through a methodology similar to an SSL/TLS handshake.

### Major Contributions

The main contributions of this paper can summarized as follows: (1) it proposes a new paradigm for authentication without the limitations of the previously proposed scheme in the literature; (2) the technique uses a hybrid, layered encryption scheme for dynamic, two-way, multi-factor verification; (3) the technique does not increase user’s responsibilities or add unnecessary difficulties for the user; (4) the scheme does not sacrifice processing speed; (5) it provides theoretical flexibility for integration with proposals set forth in other studies for combating other forms of cyber-attacks; and (6) the scheme does not create new vulnerabilities or weaknesses to other types of attacks, such as brute-forcing.

The rest of this paper is organized as follows; Section 2 provides background into related work in the field and introduces the significance of this study in relation to others, Section 3 discusses different types of cryptographic schemes and why a hybrid cryptographic scheme was chosen for our scheme, Section 4 discusses the dynamics of the proposed scheme, Section 5 evaluates the theoretical method versus select alternatives, Section 6 discusses results taken from a proof of concept and compares it to select alternative schemes that have been previously proposed, as well as discusses viability based on that comparison. Conclusion and future remarks are presented in Section 7.

## 2. Related Work and Significance of This Study

The widespread consensus on the flawed nature of username/password-based technology despite its wide application is very well known, even in the public conscious outside of academia. The rapid growth of the digital economy, and its continued balancing alongside real-life applications, has created a greater concern for authentication security than ever before, but authentication norms have been very slow to change regardless [1,8,9]. Rather than fundamental alternatives being implemented, most ecosystems have simply padded their authentication processes with additional factors. This trend can be seen in the rise of two-factor or multi-factor authentication, colloquially known as 2FA and MFA respectively [10,11,12,13]. However, the rise of padding front-end authentication portals with such techniques has led to inflated user responsibilities without as great of security as is thought; these methods help protect against front-facing brute-forcing and dictionary attacks, but often do not add any additional layers of protection to more common forms of cyber-attacks, such as phishing, man-in-the-middle (MiTM), or replay attacks [7,8,12,14,15].

Authentication is typically predicated on the concept password, whether text-based, or otherwise. This is generally sub-categorized into authentication by factors. These factors generally include: (i) something a user knows, such as PIN or alphanumeric password; (ii) something a user has, such as a key card or file; (iii) something a user is, such as biometric information (commonly, fingerprints; and less commonly, iris-recognition); and (iv) something a user does, such as drawing a signature or a pattern. Some have proposed a fifth factor, somewhere a user is (authentication based on geographic data), but this has not seen much exploration [6,10,16]. The first factor listed above tends to the most common implementation, while the aforementioned 2FA and MFA methods extrapolate such to include the respective amount of factors. While alphanumeric passwords are still the most common, they are also the most susceptible types of cyber-attacks. In most cases, static passwords are used, stored in hash or salted hash formats which an input is compared to. While these multi-factor methods may help obscure access control, they do not always prevent information, such as the password or password-hash itself, from being stolen. Since users tend to re-use passwords across websites, this threat still exists as not every website has implemented 2FA/MFA protections [10,11,17,18,19].

The vast inflation of the average number of accounts a user maintains, and in turn the sensitivity of information stored on these accounts, has also led to the creation of issues with passwords that are unrelated to the methodology of authentication, but must be taken into account when implementing secure authentication systems [9]. Users are considered to be the ‘weakest link’ in any secure system, because of the high rate of user error and balance of user responsibility with user risk. For example, users often reuse passwords or often pick weak passwords, and are also susceptible to malware, keylogging, phishing, or attacks like MiTM because of lack of technical knowledge or awareness [6,7,9]. While this can be combated to an extent, it often has to be considered alongside the inflation of user responsibility. For example, while minimum password complexity or randomly-generated passwords alongside password managers have been implemented before and have been shown to increase relative security, they also led to the creation of new problems, such as users being locked out of accounts, or storing them in insecure places in order to remember them. Password-strength metrics continue to increase, but this emboldening of minimum password strength has happened at the same time as increased processing power, and continued daily password-database leaks [6,7,10]. As a result, brute-forcing and dictionary cyber-attacks continue to be significant threats regardless. MFA methods have been implemented in tandem, but general consensus has been that MFA is not a perfect mitigation, not only for its only partial protection against intrusive cyber-attacks, but also because of additional MFA factors not being as secure as they are commonly thought to be. For example, a 2016 NIST study showed that SMS verification in 2FA is not actually secure [12]. Thus, most modern methods of security have both inflated user responsibilities and provided only an illusion of greater relative security than their simple password-authentication counterparts.

Much research in the field has been dedicated to finding alternatives to typical username and password paradigms, but little of this research has managed to find implementation outside of academic and/or research-based circles [8,16]. While proposed solutions have varied significantly, countermeasures that are derivative of current paradigms are more widely agreed upon. Beside the previously mentioned user education and minimum password requirements, typically-accepted countermeasures include tokenization, or shift from ‘static’ passwords to ‘dynamic’ passwords, generally presented in a similar fashion to MFA PINs [11,12,17,18,19]. Other countermeasures include reactive password checking, heightened access control, stronger password hashing and salting, and complex, generated passwords [8,16]. Most of these countermeasures are focused on padding the security of alphanumeric passwords however [15]. A large body of research has been done in an attempt to replace these as well; the most common of these proposals include either biometric or graphical alternatives to alphanumeric passwords [8].

A significant percentage of research on biometric authentication has stated that the quantification of its security versus alphanumeric passwords is hard to draw a decisive conclusion on. It is generally agreed upon that biometric authentication is still a flawed system [9,10,20,21], both because of false positive rates as well as privacy concerns. Additionally, the scale of biometric deployment is questionable [9,21], with it only being viable on certain devices, such as phones with fingerprint sensors as opposed to home PCs, other phone models, or even tablets. Biometrics also risk the theft of highly sensitive personalized information, something which alpha-numeric passwords do not suffer from [9,10,21]. Biometrics cannot be brute-forced/dictionary-attacked however in the same way that passwords can, and for localized authentication can sometimes be considered more secure. However, it is generally agreed upon that biometrics fail when faced with deployability and scalability [20,21]; especially since, often, the usage of biometric data requires the inclusion of additional nodes in the authentication ecosystem, such as the inclusion of an “identity provider” and a “rely party” alongside the client agent instead of a simple client to server structure [20,21]. Fingerprint authentication is mostly commonly used for biometric authentication, while other forms of biometric authentication have been studied [21], they face the same issues as fingerprint authentication.

Another alternative to alphanumeric passwords is graphical passwords [8,11,22]. There has been a degree of consensus regarding their viability, with a minor agreement that they outperform alphanumeric passwords [8,11,22]. Graphical passwords are typically sorted into three categories; recall, cued-recall, and recognition. Methodology can range from drawing an image (e.g., a signature), clicking or ordering images, selecting points on a given image, or various other graphical methods [22]. Often these systems rely on highly specific software dependencies which are not always viable for wider implementation. While graphical passwords may circumvent many issues native to alphanumeric passwords—such as brute-forcing, key-logging, and dictionary cyber-attacks—through its introduction of CAPTCHA-based elements, graphical passwords introduce their own set of native issues [8,11,17,22,23,24,25]. Primarily, deployability of graphical passwords is a continuous topic of debate; while it is usually accepted for ease of implementation on mobile devices, their implementation on web browsers or desktop systems has received scrutiny [8]. Depending on the category of graphical passwords implemented, inflation of user responsibility is another serious concern, especially for higher security patterns [8,23,24]. Much like biometric passwords, graphical password systems also face the threat of false positives [17,24]. Additional issues include intelligent-guessing, shoulder-surfing, heuristic-attack methods, and phishing [25]. Graphical passwords also do not inherently protect against cyber-attacks such as MiTM attacks [8,22]; however, studies have shown that paired alongside 2FA/MFA techniques, vulnerabilities are arguably mitigated at a higher rate than alphanumeric-passwords [11,25].

Various other solutions have been proposed, but have not received as much attention; this is typically because they aim to solve a highly specific issue rather than propose a scheme which is holistically more secure. A notable one is a popular authentication alternative named honey-encryption, a style of encryption taking its namesake from its parallel to ‘honeypot’ cyber-security techniques. Honey-encryption is a method of preventing brute-forcing and similar front-end cyber-attacks through encrypted authentication which natively returns false positives to attackers and creates alerts upon such an occurrence [26,27]. While honey-encryption has rendered favorable-test results in a vacuum, it is not generally considered holistic enough in its protections to merit replacing current paradigms. In many proposals, in order to better protect against attacks other than brute-forcing, it requires restructured and expanded ecosystems, which results not just in higher maintenance but also a greater chance of system risk [26,27].

Similarly, structural ecosystem risks are common in many of these proposed techniques; for example, a number of studies propose blockchain-based architectures [28] as theoretical authentication alternatives. However, these proposals often do not take into account realistic deployment or scalability expectations; most do not provide a proof of concept either. Other proposals often fall into a common pitfall of advocating for open-source or proprietary software, or some form of integration of such software into the authentication ecosystem [13,29]. While these studies sometimes do show improved security, alongside proof of concept, to some degree, the recommendation of highly specific and/or proprietary software as a replacement for a protocol or scheme is not completely practical [7,18].

Other research has focused on mitigating authentication concerns outside of the typical user login environment, and thus cannot generally be extrapolated onto the wider field outside usage within their specific specialized scenarios. Such research includes the usage of ‘smart cards’ or similar physical objects as a form of user identification [18,30]. Often this approach is either paired with (in a MFA manner) or as an alternative to, biometric authentication. Besides being only useful in highly specialized scenarios, such techniques are highly susceptible to theft, user error, denial of service (DoS), and insider attacks [30]. They also, like biometric authentication, inherently lack user anonymity. Similarly, they are not considered highly scalable because of these factors [18,30].

If we are to focus on research which meets the requirement of deployability and scalability, especially as an extension of current norms, rather than a complete structural and architectural replacement of it, then there is a select set of agreed upon countermeasures which can be used as a springboard [16]. First, one such measure is the use of dynamic passwords [17,18]. Dynamic passwords, also known as ‘one-time passwords’ (OTPs) are a tokenized solution to circumvent common security issues such as MiTM attacks. OTPs are considered one of the strongest methods of user authentication and provide very high security at a low computational cost [12,13]. They prevent MiTM and replay attacks in theory, but because of their relative technical complexity (especially in regard to key distribution), they are typically only implemented as a secondary factor in 2FA and MFA techniques, such as in the PIN system [12].

Another of these countermeasures is two-way verification; a method of verification by which a server does not only authenticate a client, but a client also authenticates the server. This measure commonly ties into the previous one, as two-way verification is often implemented as a way of creating dynamic OTPs [12,18]. However, two-way verification is not limited to this, and can also be applied in a more general manner to ensure mutual client–server integrity [11,18]. Many OTP-based protocols are susceptible to masquerade attacks however and are linked specifically to certain input types [12]. Other studies which have proposed two-factor derivative verification systems for dynamic logins do not implement hash-chain protocols, and thus are often still at risk of MiTM, sniffing, replay, or similar cyber-attacks [17]. Some studies estimate that dynamic passwords implemented alongside 2FA/MFA would result in the highest possible security compared to other alternatives, but it has not been aptly quantified [19].

Two-way verification is often implemented through a scheme called ‘hash-chains’. The usage of hash-chains in authentication is not necessarily a new concept, and using them for generating dynamic OTPs has been theorized before [31,32]. The oldest conceptualization of this technique stems back to 1981 [32]. However, hash-chain based schemes in the past have faced significant weaknesses. Recent research has shown that previous attempts at implementing hash-chain based authentication are still susceptible to cyber-attacks, particularly MiTM and replay attacks [32]. More evolved versions have surfaced recently, but have not penetrated the mainstream. Most recent iterations rely on highly specific infrastructure and synchronization between client and server, especially for recording timestamps [31,32]. They also often intersect with smart-card based technology [32] rather than being applied independently in a widely deployable manner for evolution of current paradigms. Various methods which have adopted hash-chains exist, including [33,34,35]. These proposals have largely implemented hash-chains for the purpose of producing one-time passwords as described above and detailed in [33], or for helping facilitate secure methods of peer-to-peer communication outside of centrally signed authorities [35].

In [33], multiple hash chain protocols are compared next to typical hash authentication means. These include Lamport’s Hash Chain protocol, YSH protocol, the Bicakci Protocol, and the HOTP and TOTP protocols among others. It was shown that protocols that used hash-chains, such as Lamport’s Protocol, were extremely resistant to eavesdropping and impersonation-based attacks [33]. Hash-chains proved to be notable in their resistance to these attacks. Protocols such as the HOTP protocol also provided tokenization, in the form of OTPs. However, issues were present in many of these protocols; for example, almost all studied protocols only provided one-way authentication, rather than two-way. Those which did manage to implement two-way authentication suffered from other issues, such as high computational complexity [33]. Furthermore, not all schemes actually incorporated cryptographic security, some only provided logical protections. The most performing protocols among of what have been proposed are the Bicakci and TOTP protocols. They provided reasonable resilience to eavesdropping, impersonation, and interception, as well as somewhat eased deployment, but both suffered from only providing one way authentication, and Bicakci’s protocol suffered from high computational complexity as well [33]. High computational complexity is an issue with many hash-chain protocols; studies such as [34] have attempted to fix this but have not found much wider implementation. The scheme described within [35] stands as another contrast to the protocols described within [33]. It provides a hybrid authentication scheme, which provides similar resilience to the stronger TOTP and Bicakci methods. However, this method is not fully extrapolatable; this is because it was built with specific focus in mind, and relies partially on physical-layer key security, which is not always available, and is not universally applicable in all networks [35]. Furthermore, the scheme in [35] also requires the use of a trusted authority node to issue tokens [35], which adds another arbitrary authority to the client–server ecosystem.

The contributed scheme in this paper is focused on building off of past schemes which have attempted to improve authentication security, specifically in this case by bolstering resistance to cyber-attacks relating to data interception, manipulation, or impersonation. While other schemes may focus on either increasing resilience to brute-forcing or dictionary attacks, this scheme instead, which is continuation of our work in [36], opts to provide a holistically secure environment for authentication transactions to take place in, while also paying mind to tangential threats such as brute-forcing, and not inflating their risks inversely.

The proposed scheme within this study aims builds off of aforementioned prior current-paradigm norms by implementing a dynamic OTP-based hybrid pseudo-hash-chain style protocol, and a strong two-way verification. The OTP (one time pad) foundation of the scheme is used to create an environment for deriving dynamic tokens, in a structurally similar methodology to hash chaining. This dynamic element is used to replace the security risk which comes from sending static data; it prevents both replay attacks as well as convolutes interception attacks as well as mitigates brute-forcing risks. Therefore, the proposed scheme focuses on mitigating the risks of more serious cyber-attacks such as MiTM, sniffing, replay attacks, and more. In doing so, while it aims to natively obscure brute-forcing and dictionary attacks, it also leaves open a flexible front-end for integration (through its adaptability as long as a front-end produces a static output which can be used as an identifier) with other proposals which are more focused on eliminating such independently. This proposed scheme aims to achieve its end goal while maximizing scalability and deployability. It also aims to build off of prior hash-chain protocols while retaining benefits of past schemes, including the resistance to middle-man or malicious entity-based interception attacks specifically, while also including additional reconcilable benefits such as two-way authentication and usage of cryptographic security.

## 3. Comparative Overview of Encryption Schemes

While there are both benefits and detriments to the applications of symmetric and asymmetric cryptographic schemes individually, we opt to use a hybrid scheme, taking strong traits from both of these separate techniques. Symmetric encryption uses a singular, shared key in order to encrypt and decrypt data sent between the receiver and sender. Asymmetric encryption uses two different keys, one for encryption, and one for decryption [37,38]. Generally, symmetric key schemes follow a uniform encryption and decryption method, sometimes in multiple or layered phases, where the same technique (or in some cases, its reverse) is applied to the cipher text for decryption and the plain text for encryption. Asymmetric encryption differs from this, using two keys generated at the beginning, related to each other, typically through a mathematical function [37]. One key is used for encryption, and the other for decryption [37,38]. Symmetric cryptography is faster, and more efficient, but harder to apply universally because of key distribution risks and management [1,38,39,40,41]; meanwhile, asymmetric encryption is more scalable [1,38,39,40,41].

Symmetric encryption is significantly faster than asymmetric, but often is not used for authentication operations because of key distribution. Since the symmetric key must be on the sender and the receiver simultaneously, transmitting the key can be a large security risk [1,38]. Asymmetric schemes are thus more common, since mathematical functions act as a detractor to cyber-attacks. However, since asymmetric schemes open themselves up to security risks by these functions being reversible or solvable mathematically, this inherently leads to brute-forcing risks [37]. This is because in most asymmetric schemes, outputs are singular given an asymmetric function can be conditionally filled. For example, if we are to look at public key cryptography, one of the most common deployments of this form of encryption, there is a public key, and if data is intercepted, then an attacker has both the public key and the encrypted message. There is only one private key which can fulfill the conditions of the function given the public key and message to produce an unencrypted message; this is self-evident within the function used to render the original encryption to begin with. While this private key may be hard, if not impossible, to find given the length of the keys involved, ever-growing technical standards as well as the possibility of re-used keys which have already been broken mean that brute-forcing is hard, but never theoretically ‘impossible’. If a symmetric key is not ever transmitted, by comparison, discerning the plain text decryption through brute-forcing is significantly harder if not theoretically impossible in some scenarios, as there is no guarantee as to its original text except for its context or place in a wider system [1,37,39]. This means that unless there is knowledge of the plaintext—or what the conditions the plaintext must fulfill—there is no readily discernible way to decrypt a symmetrically encrypted message without brute-forcing every combination of characters from each length up equally the total message size.

The hybrid method used in this scheme draws from elements of both symmetric and asymmetric cryptographic schemes. The full explanation of the techniques employed is broken down in Section 4, as they appear throughout the logistical explanations of the scheme. Hybrid encryption was employed for retaining the efficiency and speed of symmetric encryption alongside the secure key distribution methodology and deployability of asymmetric encryption [37]. Furthermore, this scheme uses what may be considered a pseudo-‘hash chain’ scheme for two-way verification and dynamic one-time password generation. Previous research has indicated that ‘hash-chains’ as a concept are consistently a more holistically-secure method of password verification [31,32]. However, previously proposed hash-chain protocols not only have high-computational complexity, but often lack two-way verification [11,18,31,32]. This scheme does not use a hash-chain in the typical sense of the word, however. While the protocol mimics a hash-chain protocol functionally, it does so in a mechanical sense rather than in a literal sense. Previous protocols have focused on hashing incremental, but high complexity, values [31,32]. Our proposed protocol exchanges incrimination for random generation, and includes a two-way handshake-style verifications based on a cyclical authentication methodology. This is done to keep the verifiable and secure nature of hash chaining but reducing computational complexity as well as deployability. Further explanation can be found in the following Section 4. Exchanging incrimination for randomization also increases the security of generated tokens by removing patterns detectable through heuristic means. Further techniques utilized in this scheme for verification also mimic that of standard asymmetric encryption techniques, using generated client and server keys based on mutual sources. Extra caution is taken in all data transactions, in order to avoid pitfalls which create attack surfaces for MiTM attack among others. In doing so, many pitfalls which existed in solely mathematically developed hash chains have been avoided. The ensuing sections provide detailed explanation and the evaluation of the proposed protocol. The actual authentication is done through a symmetric key which is generated through asymmetric means. Although symmetric encryption is used throughout the protocol, the symmetric keys are generated through functionally asymmetric means in order to make sure sensitive data itself is never transmitted from client to server or vice-versa.

## 4. Dynamics of the Two-Factor Hash Verification and Authentication Scheme (TFHVA)

**Figure 1:**
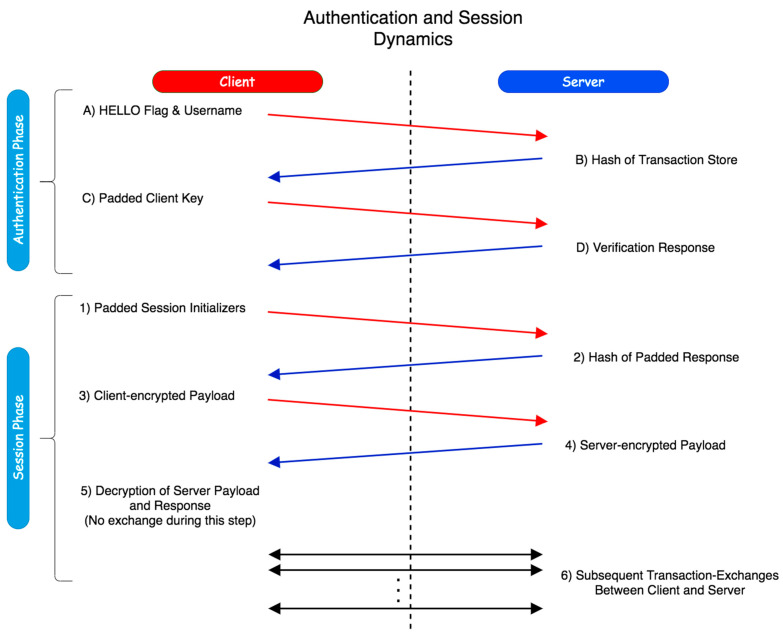
Dynamics of the proposed model.

**Figure 2:**
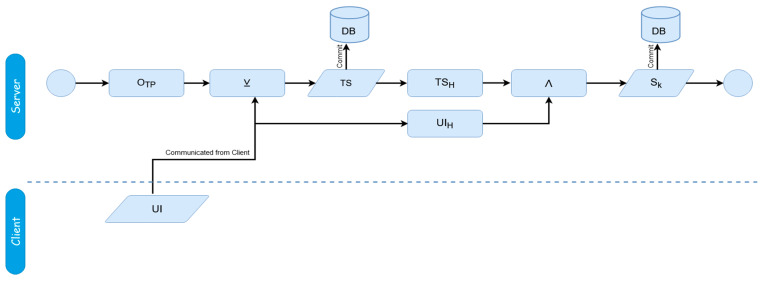
Registration Process Phase.

**Figure 3:**
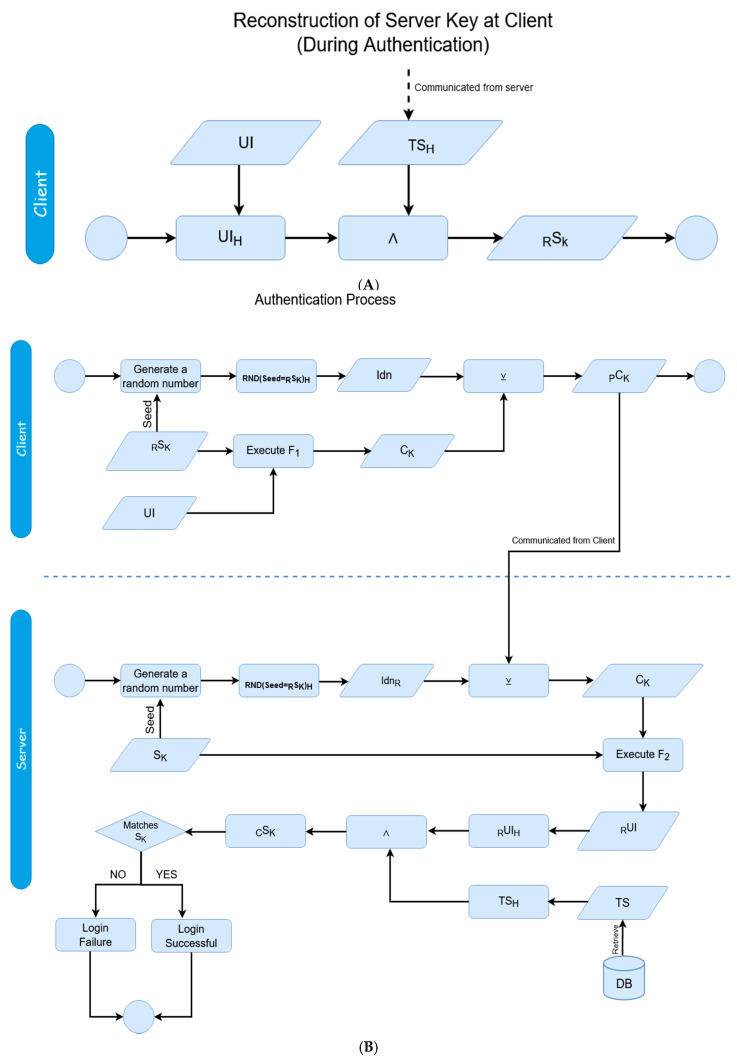
(**A**) User verification process—reconstruction of server key; (**B**) User verification process.

**Figure 4:**
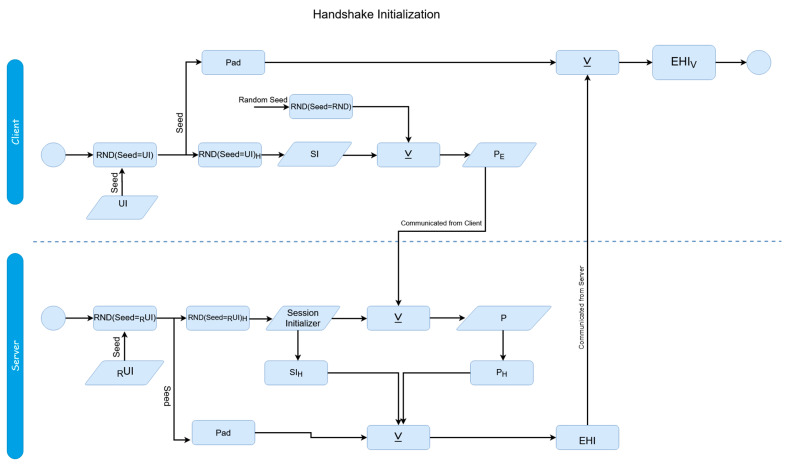
Initialization at the beginning of a session.

**Figure 5:**
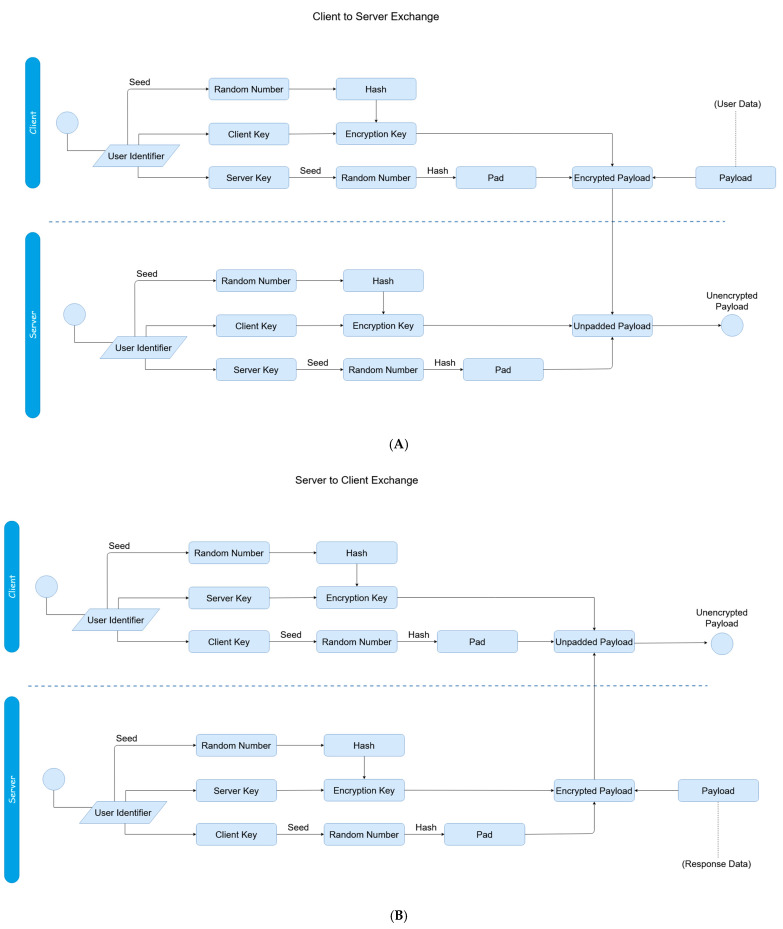
Transaction protocol, for client to serve (**A**); for server to client (**B**).

The general framework of the dynamics of the proposed scheme is illustrated in Figure 1. Two phases constitute the proposed authentication scheme; the user authentication itself, and then the session establishment afterwards. Figure 2 and Figure 3 display the mechanics of the user authentication phase, while Figure 4 and Figure 5 demonstrate session establishment. The session establishment phase is predicated on the recognition of the user authentication phase having happened as a precursor; the session establishment phase is not meant to be explicitly holistic, but rather an example of how the authentication scheme can be further extrapolated. The uniform portion of the authentication scheme, the section intended to challenge current authentication paradigm norms, is outlined in the first phase, which is shown in Figure 2 and Figure 3.

Before we go any further, we need to define some important terms and introduce some notation as in Table 1 below. The term ‘User Identifier’ is referenced in many of the diagrams and explanations as the starting point for the methodology. In this context, a ‘User Identifier’ refers to any type of data which a user can reliably reproduce, and can be converted to and from hexadecimal (thus binary) format. The length of the User Identifier is arbitrary, but as with other means of authentication, the longer the length, the more secure it is. Examples of what constitutes a User Identifier can include a key-store file, a hash from an alphanumeric password, a string derived from a fingerprint or smart card, among many other things. The User Identifier can also be the output derived from the input from a front-facing authentication system which uses biometrics or graphical passwords. The User Identifier is represented in equations and diagrams as UI, while reconstructed User Identifiers are represented using _R_UI. For the purpose of creating a proof of concept of this process, SHA256 was used as the standard hashing algorithm for producing such hashes. However, this methodology is not dependent on any specific form of hashing, so ‘hash’ may refer to any securely-equivalent hashing algorithm as scalable for individual security needs. Values used in this process are assumed to be convertible between string (i.e., alphanumeric) and hexadecimal/binary. Bitwise operations are performed on the binary representation, where any mathematical function is assumed to be a hexadecimal representation of the value.

As a further note, this section only covers an overview of the process itself—all analysis, evaluation, and security comparisons are covered instead separately in the below Section 5 and Section 6.

Figure 1 provides a general overview of the interaction between the client and server. The first phase that takes place is the authentication establishment, essentially the login, while the second phase takes place after authentication has successfully happened if desired. Both phases are expanded on shortly. For the sake of simplicity, user-registration is assumed to have already happened, and while not shown in Figure 1, we begin by going over it next. Figure 2 represents the registration phase, which occurs one time before any authentication measures can take place.

The four steps of the authentication phase (Figure 1, steps A through D) are represented in Figure 3A,B. *Step A* is the “HELLO” flag with the username being sent to the server. *Step B* is the response from the server with the hash of the transaction store (TS_H_) for the associated username from the secured database. *Step C* is the client responding with the padded client key (_p_C_k_) to the server. Finally, *Step D* is the verification of the user from the received padded client key. The regeneration of the user’s transaction store (TS) and server key (S_K_) happens after a successful verification has occurred. While the events of Figure 2 are exclusive to registration, it is important to note that as the transaction store is recreated at the end of each success authentication, and thus the events of Figure 2 are replicated to recreate this data, but with the _R_UI rather than the UI as transmitted directly from the client.

The initial authentication session is established in this process with the first two transactions between the client and the server always following identical first steps. First, a client sends a “HELLO” flag to the server alongside the associated username of the user to be authenticated. The HELLO flag exists to differentiate sending a username by itself from other arbitrary commands; if not for the “HELLO”, a username could be potentially confused by the server with arbitrary data, which could be a security risk. The server responds to this flag with the hash of the transaction store (TS_H_), which is retrieved from a local database with the value associated with said username. In the second transaction, the client sends the actual authentication request to the server using the padded client key. For the sake of organization, further information is explained for the login phase following the explanation of registration.

Figure 2 demonstrates the registration process. The user registration process must take place for the user to be able to authenticate, just as with a traditional registration/login scheme. A user registers minimally with their username and their UI, which is the data used for authentication in place of a password. Any method can be used which generates a static output, since the UI must be consistent. This time of registration is the single and only time the UI is sent to the server. This is assumed to happen over SSL/TLS to ensure security; by not resending the UI past this single point, the UI can be seen as unique data and thus harder to differentiate from other packets if intercepted. Still, this singular transaction during registration is the only point in the lifetime of this process which could possibly pose a weakness, assuming the secure wrapper was compromised. However, as this sensitive data is never transferred again, and this only takes place one time during registration (or, once more if a user changes a password), the authentication is still more secure than current paradigms, which either exchange the sensitive data with each login attempt, or submit during registration at least once regardless without the additional interceptive protections provided during the login phase post-registration.

During this registration phase, after the UI is submitted, the transaction store is created. The transaction store is derived as:

TS = UI ⊻ O_TP_ (1)

The creation of the transaction store is facilitated through the employment of symmetric encryption. It is created by performing an XOR operation; which is a symmetric encryption operation; of the UI with a randomly-generated one time pad of an identical length. A one-time pad (O_TP_) is used for two reasons; firstly, to make sure sensitive data is never explicitly stored, and secondly, to create a key file which can be used to emulate hash-chaining which produces a distinctive output. Given this symmetric encryption technique, as the OTP is of the equivalent length and randomized, the UI can essentially be considered unbreachable by brute-forcing, even in case the data were to somehow get leaked.

After the transaction store is created, the server key is created next; this is done through a bitwise-AND of the hash of the user identifier (UI_H_) (which is retained only temporarily before being discarded at the end of the registration process) and a hash of the newly created aforementioned transaction store.

S_K_ = UI_H_ ∧ TS_H_ (2)

This bitwise AND is done because AND is a destructive operator, and thus does not leave binary information about its factors and cannot be reversed. Like mentioned earlier, the UI used in creating the server key is a locally-cached copy that will be discarded once registration is done to prevent any security violations. Caching the UI for this short while prevents the need of communicating it again from the client.

Both the server key and transaction store are the only data stored in the server’s databases, both of which are tied to a user’s username, so they can be retrieved. In both the above and below figures, there are references to two variations of Hash Outputs; TS_H_ and UI_H_/_R_UI_H_. The differences in hash functions refer to pairings of functions which always coordinate to the same inputs and outputs respectively. Since the hash functions are essentially left up to the user, any form of hashing can be used, but the same function used in TS_H_ must be used universally for all instances of *TS_H_*, and the same with UI_H_/_R_UI_H_.

We now begin the explanation of the core authentication method. At the time of requesting authentication, the hash of the stored transaction store is sent to the client. This is essentially a dynamic token, and where the pseudo-hash chaining portion of the protocol comes into being. A hash-chain is a technique for derivation of multiple one-time keys from a single hierarchical piece of data. Through the use of tokenized data originating on shared mutual secrets (in this case, the UI), a methodological chain of derivative keys is created. This tokenized aspect is foundationally what increases the security of the protocol; as only tokens are exchanged, small bits of data which only make sense when combined with locally equivalent data only known by a mutual client–server pair, the risk of interceptions are significantly decreased, and sniffing-based attacks become meaningless. Even if data is intercepted—whether every transaction or one individual transaction—the data is meaningless without also containing the locally stored data of client and server, as the tokens only signify how the data on both sides are to be respectively manipulated. Furthermore, as the tokenized data is derivative of a one-time pad, risks for attacks such as replay attacks are also greatly reduced; the dynamic element of this is explained further on. Impersonation attacks are also reduced, as multiple, essentially randomized elements (due to the randomization of the OTP used in creating the transaction store) would need to be generated to impersonate a server, and data which fulfills a number of arbitrary conditions derivative of a token (which are never explicitly hinted at by the client or server during a transaction) would need to be guessed on the side of a client. This is also where the ‘two-factor’ nature of the scheme comes into play, as because of this, the client and server validate each other, the server does not simply validate the client alone.

As the client has or can reproduce the UI on their end locally, the client can derive an equivalent server key (the derived server key - _R_S_K_) through a bitwise-AND of the local-equivalent hash of the UI (UI_H_) and the received hash of the transaction store, as shown in Figure 3A. The client can then derive the client key (Ck); this is a token which can be functionally factored with a server key in order to reconstruct a user identifier (_R_UI). The process of deriving such follows logic parallel to a hash-chain; using a shared secret from both participating parties, hash data can be derived which only has meaning upon exchange to the opposite party—in this case, this data is each party’s respective ‘key’—the client or server key. In this case, the hash is not linearly iterative (as in, there is not a shared counter of 0,1,2, and so on) being kept on either side as they typically are with chains, but rather an algorithmic function stemming from randomized elements. As the shared secret relies on the mutuality of the user identifier, the OTP added to the transaction store, which injects the randomized element can be seen as the functional means of producing a ‘chain’ for deriving temporal data from a singular otherwise shared source. This client key is derived from the local user identifier using any function whose inverse is defined; in other words:

F_1_ (UI, _R_S_K_) = C_K_ (3)

F_2_ (C_K_, S_K_) = _R_UI (4)

The function itself is arbitrary so long as it can be inverted with the same inputs and outputs shown in the F_1_ and F_2_. In this case, for proof of concept, we used simple division and multiplication; C_k_= UI × S_k_, and thus, inversely, UI_H_ = C_k_/S_k_. In general, however, any function whose factors can be inverted can be used to scale this methodology for equivalent security needs. This process can be viewed as a form of asymmetric encryption, by which two keys are used to functionally produce a third key.

The client key is the second transaction from the client to the server, as shown in Figure 3B. Before the client key is sent however, it is XORed with a pad, referred to as the ‘Identifier’ (not to be confused with the ‘User Identifier’). These pads work through the fundamental basis of symmetric key encryption. This is done for two reasons; firstly, so the value of the client key is further obscured, invalidating data which could be stolen by an eavesdropper, and secondly, to verify that the server has not changed. As the only way to decrypt the client key is by having an equivalent server key which both the client and server maintain, if a server can decrypt the client key, then they can be said to be the same source the authentication process started with, thus subverting any possible masquerade attacks. The value of the ‘Identifier’ (Idn) is produced from using the server key as a seed for a pseudo-random number generator, and taking the hash of the produced value.

Idn = RND (seed = _R_S_k_ )_H_ (5)

_P_C_K_ = C_K_ ⊻ Idn (6)

The padded client-key above made from the XOR of client key and identifier (Idn) is sent to the server. The server then can decrypt the received data to receive the value of the original client key, see bottom part of Figure 3B:

Idn_R_ = RND (seed = S_k_ )_H_ (7)

C_K_ = _P_C_k_ ⊻ Idn_R_ (8)

Now that the client key exists on the server, and the server has the local server key, the server can reconstruct the user identifier through the inversion of the function used to originally find the client key (the F_1_ aforementioned as opposed to the F_2_ used to create it). This inversion can be looked at as the asymmetric companion to the prior asymmetric methodology used to derive the sent data to begin with, again using two inputs to functionally derive a third input from encrypted data. In this specific case, since we used multiplication (UI × _R_S_K_) to find the client key in F_1_, we now use the inverse operation – division (C_K_/S_K_) to find the user identifier. The server now has the reconstructed User Identifier.

From this reconstructed user identifier on the server, the final step of verification can begin. The hash of the constructed identifier is taken and is combined through a bitwise AND with the hash of the stored transaction store. The value of this combination is then compared to the stored server key; the authentication is considered a success if the values are equivalent to each other. Otherwise, it is considered a failure. This cyclical methodology ensures the original data constituting the shared secret does not need to be sent again, and another randomized OTP can continue the process of randomized pseudo-hash chain key derivation.

At the end of each successive authentication phase, the transaction store and server key are replaced, as to add an element of dynamics to the methodology, which was previously mentioned. This dynamic element invalidates any prior data which could have been sniffed or saved, adding an inherent resilience to spoofing and replay attacks. Since the user identifier is constructed on the server key in order to verify the user, the reconstructed user identifier is XORed with a new one-time pad in order to produce a new transaction store replacement. A new server key is then generated through the same process which it was at registration, with the bitwise AND of the hash of the newly generated transaction store and the reconstructed user identifier. The reconstructed user identifier is then scrapped.

For the purpose of simplicity, a simplified breakdown of each authentication step can be seen below, alongside a short explanation

1. Server
  (a) TS = _R_UI ⊻ O_TP_
    - Above step happens prior to authentication but is included for clarification. Transaction store, TS, created from the one-time-padded user identifier (X)
  (b) S_K_ = _R_UI_H_ ∧ TS_H_
    - Same as the above, this happens prior to authentication but is included for clarification. The server key (S_K_) is created from the hash of the user identifier and transaction store. The local user identifier is scrapped.
  (c) TS_H_
    - The hash of the transaction store is sent to the client.
2. Client
  - _R_S_k_ = TS_H_ ∧ UI_H_
    - Having received this from the server, the client can create the local copy of the server key
  - C_K_ = F_1_(UI,_R_S_K_)
    - The client key is created from through an output function with inputs of the local user identifier and the received server key.
  - Idn = RND(seed = _R_S_K_)_H_
    - Identifier is created using the local server key as a seed
  - _P_C_k_ = C_k_ ⊻ Idn
    - Padded client key is created through an XOR of the client key and identifier. This is sent to the server.
3. Server
  - Idn_R_ = RND (seed = S_k_)_H_
    - The server can recreate the identifier using the local server key.
  - _R_C_K_ = _P_C_K_ ⊻ Idn_R_
    - The server can unpad the received client key using the generated identifier and the received key.
  - _R_,UI = F_2_(_R_C_K_, S_K_)
    - The server can regenerate the user identifier by using an inverse function with inputs of the client key and the local server key.
  - _C_S_k_ = _R_,UI_H_ & TS_H_
    - The server recreates a constructed server key for validation with the reconstructed user identifier
  - S_k_ == _C_S_K_
    - The server validates the recreated server key
  - TS = _R_UI ⊻ O_TP_
    - Upon validation above, the regenerated user identifier is used to recreate the transaction store (essentially step A, but here for clarity). The old transaction store is discarded.
  - S_K_ = _R_UI_H_ & TS_H_
    - Upon validation above, the regenerated user identifier and new transaction store are used to recreate a new server key (essentially step B, but here for clarity). The old server key and the regenerated user identifier are both discarded.

With the initial authentication verification stage of the protocol having taken place, the previously mentioned phase two, the session protocol, can be invoked to securely transfer data between the client and server, as shown in Figure 4 and Figure 5.

In the case that a session protocol is initiated, the transaction store swap would occur at the termination of the session rather than the end of authentication. While this is best used alongside a layered outer encryption protocol such as SSL/TLS to provide a layered digital envelope method of transfer, this session methodology could theoretically stand-alone without such in place.

Before a session begins, a handshake between the client and server must take place for establishment; similar to how a handshake is utilized in the SSL/TLS process. This is done not just for initialization purposes for the scheme, but also to make sure neither the client or server have changed, as an attacker might be attempting to perform a masquerade attack. Prior to the first transaction, the client and server both generate respective pseudo-random numbers using the user identifier as the seed (the server reconstructs the user identifier during the authentication phase as previously mentioned and caches it locally). A new hash is taken of these numbers (all instances must use the same hash function, where the input is always the session initializer. In the above Figure 4, this is referred to as ‘Execute Hash Function’); these are the ‘session initializers’. The interaction of elements for the protocol can be seen in Figure 4.

At this stage, the process is depicted in the bottom half of Figure 1. During Step 1, the user generates the session initializers, and also generates a random number using a random seed; the “random seed” shown within Figure 4 is itself randomly generated, and only used for the purpose of generating another random number. This is done to ensure that the random number used is completely random and not using any prior cached seeds. It XORs these values together, and sends the result to the server; this is referred to in the above Figure as a ‘payload’ (specifically the ‘encrypted payload’, since this contains a pad). The server decrypts it by XORing the received value with its own session initializer. During Step 2, the server sends the encrypted hash identifier back to the client:

EHI = P**_H_** ⊻ SI_H_ ⊻ Pad (9)

These XOR operations act as a means of employing symmetric encryption to encrypt the identifier through a shared key. The pad is generated by using the originally generated number for the session initializer as a seed itself, and then double hashing the output from the pseudo-random number generator:

Pad = RND (seed = (RND(seed = _R_UI)_H_)_H_ (10)

The reason the padded, hashed value is sent back and not just the same text sent over is for a similar reason that the client key is padded during the authentication process. Firstly, so no transactions are duplicated, making sure as not to create a security risk in case of eavesdroppers, and secondly, if the hash was not taken of the value, it would essentially be the same value being send back again, as the pads would be derived from identical sources, making the process an otherwise meaningless exchange of the same value (which would be easily predictable). On the client’s side, the client can recreate the pad themselves through an identical process as the server, and XOR this value with the received data in order to find the unpadded result. The client then compares the hash of this value with the hash of the originally sent value to verify the handshake with the server.

The initial handshake is now complete, ensuring that the remote source, the server, has not changed, thus subverting possible masquerade attacks, and both sides are not being impersonated. With that complete, the session protocol is now detailed in Figure 5A,B; these next steps refer to Steps 3–6 of Figure 1 additionally.

In Step 3, the hexadecimal representation of the hash of the previously random number taken from using the user identifier as a seed is multiplied with the client key, and then a hash is taken of the resulting value; this is now the encryption key. A pad is generated using the hash of the server key as a seed for which a random number is generated through a pseudo-random number generator, whose hash is then taken. The encryption key is XORed with the pad which is further XORed with the desired payload to send to the server. In Step 4, the server received the encrypted payload. Since the server has the server key and still has the reconstructed user identifier, it can reconstruct the client key, and also therefore reconstruct the encryption key. It can also recreate the pad since it has the server key. The pad and encryption key are both XORed with the received encrypted payload, and now will have the unencrypted payload. This described process refers to the Figure 5A.

When the server wants to respond with sending payloads back to the client, the server can undergo the same process through which the client had created the encryption key and pad, but swapping the server/client key’s place in the respective creation of each element. The client then receives this response in Step 5, and can follow the same process of reconstructing the encryption key and pad to unencrypt the received payload. This can be seen in Figure 5B.

Finally, Step 6 represents any subsequent transactions between the client and server, which continue using the respective constructed keys and pads. As reusing the same encryption key and pad could pose a security risk due to either clear text, comparative, or heuristic attacks, the client and server should either rotate or re-generate keys after each two way transaction; this can be achieved using hash chains, as both the client and server know what previous value and hash generation were used as the original base value to create the encryption key(s) and pad(s). As such, with incremental, rotational, or similar counters, hash chains can be used for ensuring further dynamic security. Once the session is over, all keys and temporary data (such as reconstructions) are discarded by both the client and the server. Different keys are used on the client side and server side for integrity; otherwise, it would be easier to impersonate a client or server by simply reusing a key if a plaintext of a simple transaction was able to be brute-forced or matched from a dictionary. By differentiating keys asymmetrically, transactional data is further obscured by making sure no symmetrical data is ever exchanged, and even the same clear text will appear differently from server to client and client to server.

## 5. Performance Evaluation

**Figure 6:**
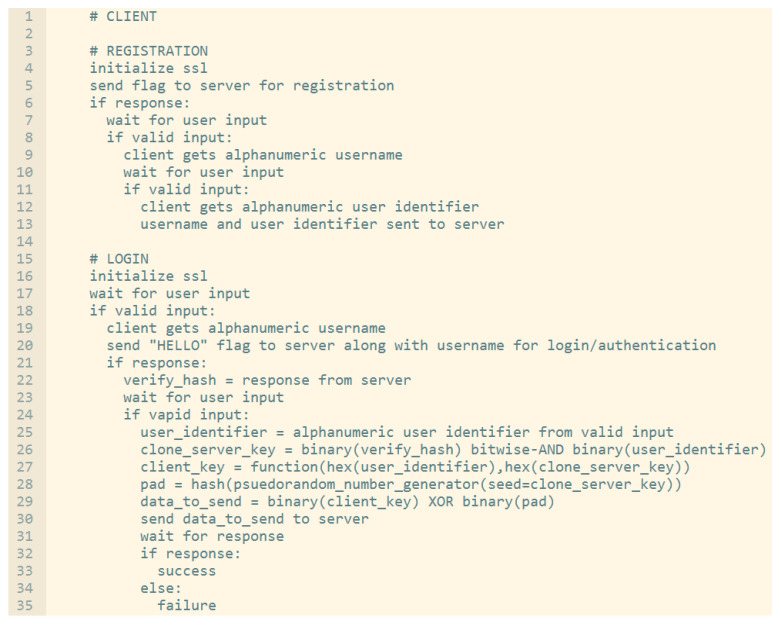
Code of client.

**Figure 7:**
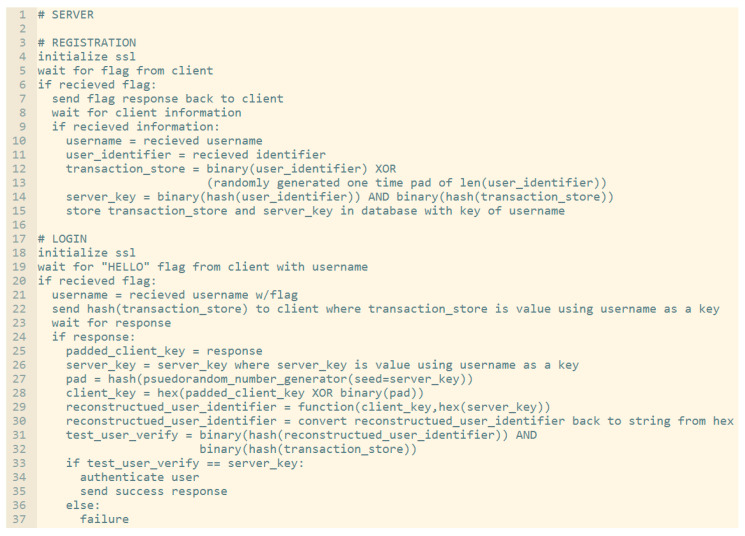
Code of server.

Before we go through the details of the performance evaluation of the proposed scheme against the other schemes, we provide the pseudocode for both client side and server side as shown below in Figure 6 and Figure 7 respectively.

In evaluating the viability of this proposal against other recent proposals and accepted paradigms, two main security vectors were looked at: first, time complexities for brute-forcing across each method; and second, non-mathematical evaluation of secure factors. In this section, we discuss this solely from the perspective of evaluating the theory; the actual results as collected from the proof of concept implementation are provided separately in Section 6.

The reason brute-forcing was evaluated, despite this scheme not being focused on brute-forcing, was to make sure the scheme did not develop a weakness to brute-forcing making it proportionally weaker to other schemes. It should be noted, as stated in previous sections, that the main goal of this paper is not to prevent brute-forcing, but instead other cyber-attacks; however, this time-complexity information is still given as a proof that the proposed methodology is on par with accepted norms in that regard. Only contemporary alternatives which retain alphanumeric-style passwords were evaluated for time-complexity. It should also be noted that, as stated elsewhere, this proposal is not dependent on alphanumeric passwords, and could easily be modified to work with other forms of input, such as graphical or biometric input. However, the alphanumeric interpretation of this method was still evaluated as such since the proof of concept was implemented in such a style.

The evaluated time complexities for an adversary brute-forcing alphanumeric can be seen in Table 2 below.

The time complexity of as password hash comparison assumes *n* is equal to the amount of possible combinations (2^x^ if *n* binary, or 62^X^ if alphanumeric (considering both lower and uppercase letters), where *x* is the number of digits) and *m* is equivalent to the complexity of one hash of one possible combination. For the attempt-based password, the time complexity is a similar O(A × n × m) where n and m are equivalent to the above; A is the number of attempts needed. However, since A is a constant, it does not factor into the overall time complexity. For the two-factor password-security, brute-forcing is slightly harder as it depends on data extracted from an image. However, it is still possible since equivalent values can be forged and sent to the server. As such, *n_1_* and *n_2_* represent the possible password and secret key respectively, while *n_3_* represents the forged data. O(m) is the complexity of one evaluation of, not only the hash of the password, but also the function of F(*n_2_, n_3_*) which produces the output derived. Finally, in our proposal, n and m are the equivalent as in the password hash comparison method. However, the introduced l is the evaluation of one function O(l) for which the hash function of O(m) is used for deriving keys. As such, the time is O(l + m) multiplied by the combination input O(n). While the work of Biswas and Biswas [38] produces a higher brute-forcing time-complexity for adversaries, it is clear that our proposal TFHVA scheme is still stronger than standardized alphanumeric password systems in this regard when alphanumeric passwords are used for the user identifier. Thus, we can conclude we have not created any additional weaknesses from this vector.

In our non-mathematical evaluation of secure factors, we looked at the functional capacities of the proposed methodology theoretically. This was a complex, multi-pronged evaluation which was done in a vacuum given the functionality of the process. Below, we compiled a list of common cyber-attacks and evaluated individually how this process protects against them.

As the purpose of the scheme was primarily resisting interception and impersonation-based attacks, we have evaluated the following common cyberattacks in a step by step manner; Man-In-The-Middle (MiTM), Sniffing, Masquerade, and Replay. Furthermore, as to prove the attack does not open additional vulnerabilities inadvertently in areas which the scheme is not primarily focused, we have also evaluated two more of the most common authentication-based cyberpunks, brute-forcing and ‘leaked credentials’, similarly.

**Figure 8:**
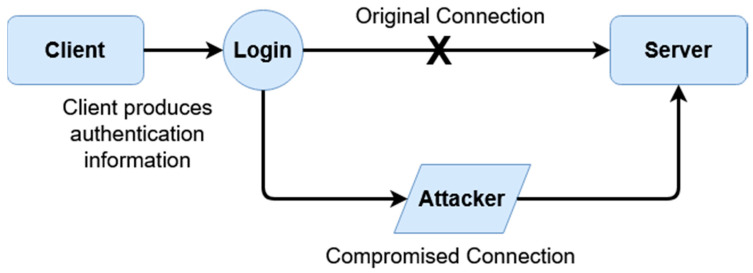
MiTM attack under a normal authentication system.

**Figure 9:**
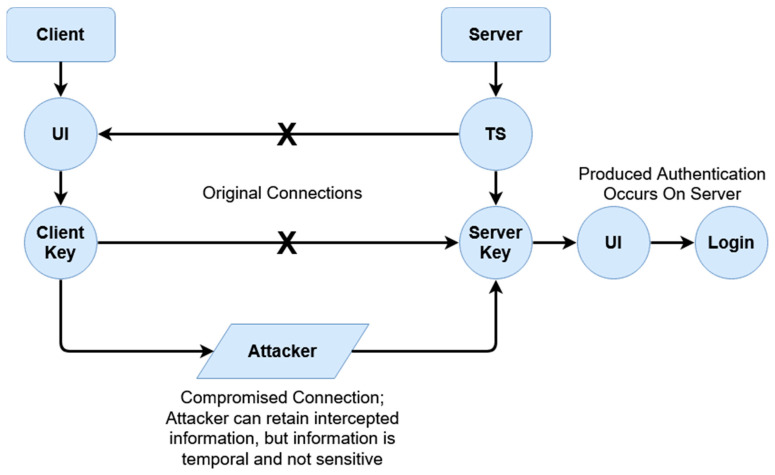
MiTM attack under TFHVA scheme.

Firstly, we are evaluating defenses against MiTM attacks, which are one of the most common interception/impersonation-based attacks. During a MiTM attack, a malicious entity acts as a legitimate connecting party, impersonating the destination or source meant to be connected to. Data is then levied through this middle-man, typically circumventing encryption such as SSL/TLS by falsifying certificates, therefore receiving access to sensitive exchanged data. In order to understand how this scheme protects against these attacks, Figure 8 and Figure 9 below demonstrate the difference between a typical authenticative connection and a connection made using this scheme.

The differences in security are rendered from the fashion in which data is transferred between entities. For most common authentication schemes, regardless of how data is input, or what front-facing mechanism (graphical, multi-factor, etc.) is used, sensitive data can be exposed if a connection itself is compromised. In this given scheme, this is averted through data being separately encrypted through tokenized derivatives of a shared secret; furthermore, as sensitive data relating to logins can be compromised as shown in Figure 8 (such as passwords or password hashes), login information seized by malicious parties when this scheme is active is temporal, and is also not indicative of value of the shared secret itself. In Figure 8, the attacker has access to all data conveyed between parties; this includes, inherently, anything exchanged during authentication, which would include the login information, hashed or otherwise. If hashed, while not exposed, information can be replayed or used to mimic the client.

Sniffing would largely occur in the same manner as a MiTM attack. However, in comparison, Sniffing does not typically attempt impersonation or interception, and instead attempts to simply monitor packets to or from a given source. Given this, the process would look near identical to the above. In comparison, however, information from a login is simply protected by whatever outer-layer SSL/TLS protections exist, while in the case of this scheme, information is encrypted with the derivative secret key (client or server key respectively), and padded with a randomized hash pad as well for further protection.

**Figure 10:**
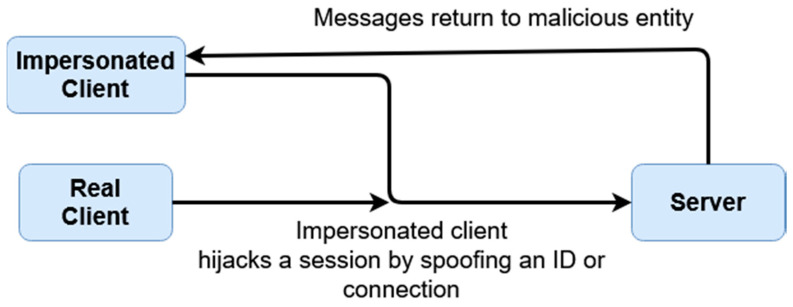
A masquerade attack under a normal authentication system.

**Figure 11:**
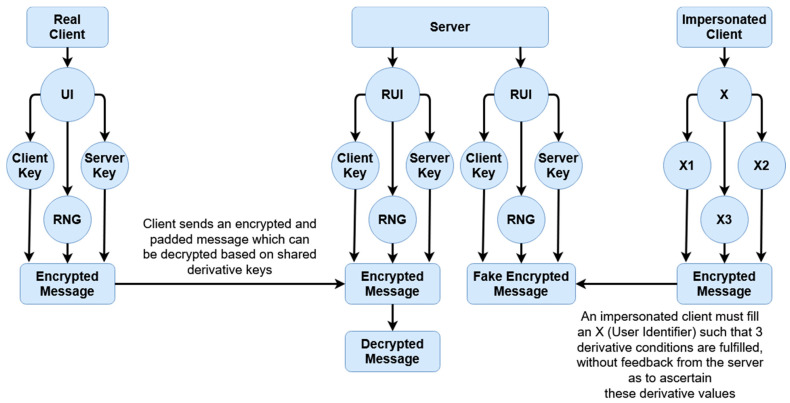
A masquerade attack under TFHVA scheme.

A masquerade attack is when a malicious party impersonates the identity of another legitimate user in order to gain unauthorized access to a system. This can be done through a multitude of ways, including hijacking of packets, IP spoofing, or other such means. The below Figure 10 and Figure 11 the differences between a masquerade attack for other authentication proposals and this scheme.

UI refers to user identifier, RUI refers to reconstructed user identifier, and RNG refers to a randomized number based on the UI seed.

This scheme is more resilient against masquerade attacks for two reasons. Firstly, data is temporal and tokenized, so it is meaningless if compromised. Secondly, within each given session, data is essentially ‘signed’ using the tokenized key of the other party. This signing means unless the attacker can brute-force the temporal token which constitutes the key, there is no way to correctly impersonate a given user. In Figure 10, an example is given of how a masquerade attack may occur. After a real client authenticates, an impersonated client may hijack the connection, redirecting the connection back from the server to receive messages as if they were the client. Because the authentication has already occurred, even if the authentication is based on access tokens, they can still receive messages back from the server even if they cannot send further connections without re-authentication. By comparison, Figure 11 shows an example of how a masquerade attack would occur within this scheme. There are at least three factors which must be fulfilled to replicate a client; the user identifier (X), and then the derivative client key, server key, and random generated number (X1, X2, and X3). These elements are all used throughout the encryption process. Even if a malicious entity is able to intercept a client key by sniffing or otherwise, they would also need to replicate X2 and X3. As these are never explicitly sent and only stored locally, these would have to be brute-forced; however, as they are attempting to hijack an active session, by the time a first falsely encrypted message is received, action can be taken by the server as the keys not matching the keys of the server (because of sharing the same original secret, the UI, as the client) would immediately create a warning.

**Figure 12:**
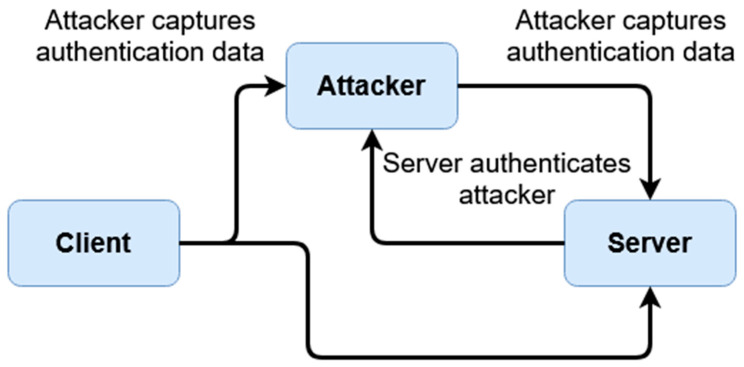
A replay attack under normal authentication pretenses.

**Figure 13:**
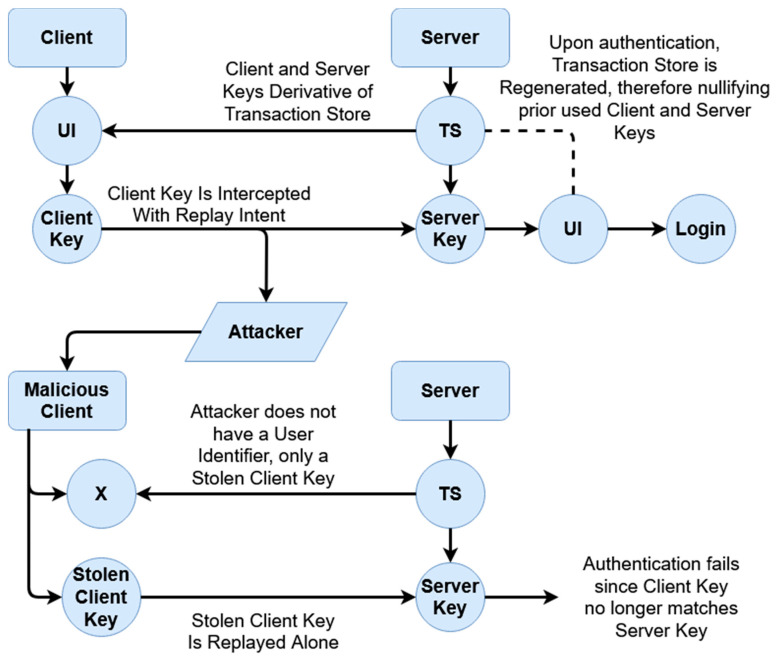
A replay attack under TFHVA scheme.

Similarly, this scheme bears a heightened resilience toward replay attacks for the same reason. As sessions are temporal and keys are derived with each session, a malicious party cannot simply save and replay packets in order to gain unauthorized access. An example can be given in the below Figure 12 and Figure 13.

The above Figure 12 and Figure 13 display replay attacks. For typical authentication, schemes that are not tokenized are at risk of replay attacks if a packet is captured, provided a third-party authentication service (such as an access token issuer) is not provided. However, these access tokens can often still be falsified if a malicious party knows a user’s username. By comparison, the tokenized temporal nature of this scheme prevents replay attacks, as seen in Figure 13. To break down this diagram, we first look at the original transaction from which the client’s packet is captured. This happens when the padded client key is sent to the server, where the authentication occurs on the server end given the received client key. After authentication, as described within the prior section, the transaction store is regenerated given the recreated user identifier, which in turn would mean both the prior client key (which was sent) and the prior (locally used) server key derivative of the prior transaction store are now moot. The malicious client does not have a user identifier (as represented by the X in the above diagram), only the stolen client key. However, as authentication has already occurred, during the ‘replay’, the newly generated server key rejects the prior stolen client key, as it is no longer compatible.

Finally, we will evaluate additional non-interception-based threats; these would include brute-forcing, and a broader ‘leaked credentials’ attack. Brute-forcing can be separated into two sub-categories; combination and dictionary brute-forcing. Combination is simply trying all combinations of a given length, while dictionary refers to running through a dictionary compilation of preset data. A ‘leaked credentials’ attack is a wider term for referring to a scenario in which a database is hacked, and as a result the data stored within is leaked; in most cases this means hashed and/or salted data is leaked alongside a given username, alongside any other stored associated data. The damage this attack can do varies. For a combination brute-force attack, we can refer to the evaluations in Table 2, specifically the first three rows. The first row applies to this scheme, while the next two rows refer to typical authentication schemes. Based on the first row, we can see that this scheme provides an above average resistance to brute-forcing attacks; therefore, we can conclude we have not opened an accidental vulnerability here. When it comes to dictionary brute-forcing, the strength of this scheme is dependent on the manner in which the user identifier is derived. Just as with any text-based input, the scheme is equally vulnerable to this attack if the original ‘password’ has been compromised as any other authentication scheme. However, as this authentication scheme is compatible with non-textual inputs—take graphical or biometric inputs—the scheme is not solely dependent on this form of input. It can be said therefore that this scheme does not create any additional vulnerabilities in this sense.

Lastly, for a ‘leaked credentials’ attack, we must look at how information is stored. If information was to be leaked from a database, a malicious entity would have the transaction store and the server key. To reiterate the above section, the transaction store is equivalent to

TS = UI ∧ O_TP_ (11)

Similarly, the server key is equivalent to

S_K_ = UI_H_ ∧ TS_H_ (12)

Because the transaction store was encrypted symmetrically using a randomized one time pad, it can be considered theoretically unbreakable given the time needed to decrypt it. However, the client does possess the server key; this is not a danger. Firstly, the server key cannot be used broadly because it was temporally generated similar to the client key. Secondly, even if the server key was used in an attempt to reverse engineer the user identifier, because of the manner in which it was created, this would pose extreme difficulty. Firstly, the attacker would need to pose data which, through an AND operation of the transaction store hash, would create the server key. This would have to assume the client knows which hash algorithm the server was using. Even if the attacker was to assume the default described within this paper (SHA256), they now face deducing the multitude of values which could result in this hash. Since AND is a destructive operation, simply having one side of the original operation does not inherently produce the other. Even if we are to assume that they can find the correct theoretical value, they still do not have the user identifier—only the hash of the user identifier, which then must itself be unhashed, which itself would follow a time complexity of O(n). By comparison, if a typical authentication scheme finds a hashed value, the time complexity is simply O(n), but the hash is immediately obvious to the hacker, there is no extra logic or steps which must be used to deduce what the value is beyond interior database encryption, which presumably would apply to any system deploying this scheme as well. Thus, we can conclude that there are no additional vulnerabilities being added through this form of attack either.

Based on the above, it can be concluded that this method provides a strong inherent defense against most common attacks on authentication, especially the focused fields of attacks resulting from interception and/or eavesdropping as intended. For most of the weaknesses and pitfalls that this methodology suffers from, minor variations can be adopted to circumvent those universally. Harnessing this process alongside other such front-facing authentication methods such as biometrics not only inherits the strengths that such has in and of themselves, but provides an additional layer of security. Furthermore, augmenting the methodology with 2FA/MFA provides yet an additional layer of security than the combination of 2FA/MFA does alongside other methodologies since the base security of this process is stronger. For the maximum amount of authentication security, a non-alphanumeric password alongside 2FA using this methodology for dynamic transactions and protections against interceptions would produce maximum resilience.

In comparison, to past hash-chain based protocols, this scheme provides several distinctions; it retains the ease of implementation (seen in pseudo-code), usage of cryptographic algorithms, resistance to eavesdropping and interception/impersonation based attacks, and also adds elements of dynamic tokenization temporarily, as well as providing two-way authentication without the usage of a middleman or separate trust authority. In comparison to the studies presented in [33], this study mimics the strengths of the best performing protocols while also providing the additional benefits mentioned above. Furthermore, it retains the simplicity of implementation as seen in the presented pseudo-code in the beginning of this section. In comparison to [35], it matches benefits and dynamization of the protocol without the need for reliance on physical layer security or a tertiary trust authority outside of the client–server ecosystem, and it is more extrapolatable.

## 6. Results and Discussion 

As seen in the evaluation section, the proof of concept for this proposal was implemented using Python to create a minimal client and server for studying results. In summarizing results, we measured time needed for authentication, security/resilience to cyber-attacks through a qualitative approach, and finally efficiency of methods through similar qualitative methods. Comparisons of such can be seen in Table 3, Table 4 and Table 5.

Reflecting on collected qualitative data, we found that our scheme provided a significantly stronger level of resilience to cyber-attacks, as outlined in the previous section as well, without requiring changes to the client–server structure, or especially increasing any burden on the user. The study was compared to similar work that does not require changes in server infrastructure, and the qualitative data was recorded in Table 3 and Table 4. Furthermore, the proposed method of two-factor hash verification and authentication scheme (TFHVA) was shown to be highly flexible—it could be adapted to support any type of data from which the user identifier is derived. However, the user identifier was the greatest variable in relative security. In this system, however, a strong password was significantly more secure than the equivalent from within the current authentication norm, password hash systems, due to the addition of safeguards against man-in-the-middle, replay, masquerade, sniffing, and other such attacks, without any structural changes to achieve higher relative security.

The greatest benefit of this methodology proved to be its strength against network, data-manipulation, and adversary-based cyber-attacks, such as, again, the aforementioned man-in-the-middle and replay attacks. It also showed a strong resilience to data leaks, and this is further emphasized by the simplicity of its implementation without any changes to existing architecture. A comparison of strengths and weaknesses of relative methods which do not require ecosystem changes can be seen in Table 4. The main resistance in this case is predicated on the two-way nature of the authentication methodology, as well as the lack of sensitive data being transferred. The main vulnerability comes from the user-registration phase, as this is the only place a user identifier could possibly be compromised. However, this is no different than any other proposed methods—as this method is assumed to work in an otherwise secure system, insecurities at login would supersede this methodology in and of itself. Furthermore, the data sent during the authentication phases are both derivative and dynamic, and thus, if intercepted, do not pose a risk in and of themselves. Furthermore, the dynamic pad on the server shows that previously intercepted data or stolen packets cannot simply be replayed. Other strengths, such as resilience to leaks, can be seen in the breakdowns of the evaluation section; such evaluations held true in the proof of concept.

Furthermore, the process is not explicitly tied to any specific hashing algorithm or mathematical function. If SHA256 is found to be insecure for example, it can be substituted with any other more secure hash function without any loss of functionality. Secondly, as this study aims to protect against MiTM and replay attacks without as much of a focus on brute forcing by comparison, it is flexible through its ability to be reconciled with other research which has namely been focused on preventing brute-forcing attacks, like the aforementioned biometric or graphical front-facing interfaces. The output of any such study could take place of the user identifier for example, which would thus increase security against brute-force attacks, and also retain the resilience to other attacks that this study is more tailored toward.

Table 5 displays average authentication times for simple skeletons of various alphanumeric methods. The proposed process was compared against two other studies, as well as a simple implementation of a password–hash comparison. Transaction times were also logged and noted. The proposed method did not sacrifice time as a trade-off for security, which other studies, such as [28] did. However, as mentioned above, this is reliant on the way the user identifier is generated—in this case, a long alphanumeric string was used. In cases where images need to be uploaded or biometrics scanned, the relative authentication time would likely be higher, and the trade-off of time would have to be contrasted with other security evaluations of this method. On the fourth column, the number of cryptographic operations used in each protocol are listed. For the most simple hash comparison scheme, only 1 operation is used, and that is the hash of the user password to be compared with the database. For the attempt-based password [42], this number is equal to the amount of times which a password is input, so the number of operations is equivalent to a variable N, once per input up until the max number needed for authentication. For the two-factor password security scheme [38], the number of operations was 2. These operations included the derivation of the secret key and the password derivation. The password derivation is the same as the other protocols in that it is just the hash of an input. The other operation, the secret key derivation, was more complex, and was based on the derivative of a secret based on a key; from either a file or an image respectively. Additional non-cryptographic operations take place as well during the key derivation process. For the TFHVA process, there were four cryptographic operations, which is the highest number on the table above, but these are quick hashing operations. Additionally, as noted in the table, non-cryptographic operations are used; these include XOR, AND, and the chosen derivation function (and therefore derivation inverse). While XOR and AND are negligible, it can be said that the derivation function is certainly variable in determining the speed of the overall protocol.

In order to further demonstrate the technical protections which this scheme offers against attacks, we demonstrated a man-in-the-middle on both a simple login system, as well as our developed scheme. The purpose of this test was to explicitly demonstrate how this scheme mitigates focused attacks. The man-in-the-middle attack was demonstrated within a local testbed. A middle-man node impersonating a server carried a falsified SSL certificate. The setup used the SSL certificate to mimic the client and pass through information to the server, and also receive information in turn and pass it back to the client. The middle-man had a simple purpose, to mimic the behavior of a theoretical attacker; in this case, circumventing SSL protections to sniff raw data transmitted between the client and the server.

**Figure 14:**
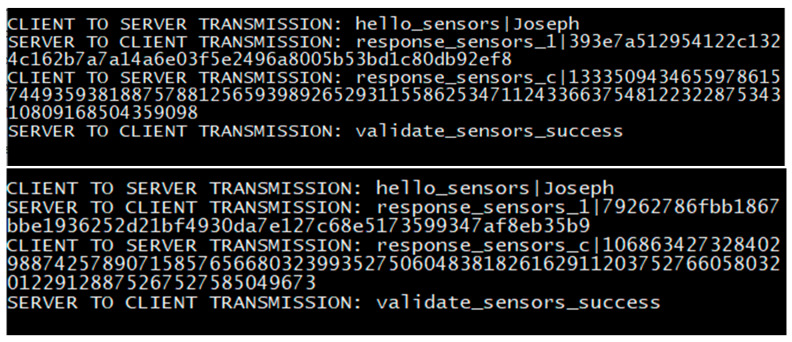
Intercepted data from client and server using the TFHVA scheme, between two different sessions.

**Figure 15:**
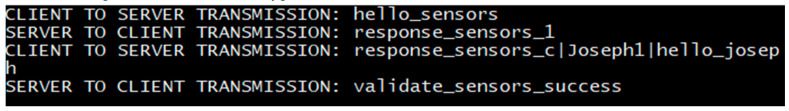
Intercepted data from client and server using a simple hash comparison login—hashing on the server side.

**Figure 16:**
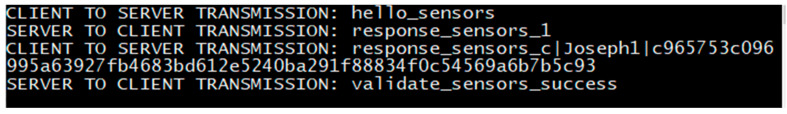
Intercepted data from client and server using a simple hash comparison login—hashing on the client side.

As an explanation of the data shown in the respective Figure 14, Figure 15 and Figure 16 below, data is exchanged between a client and server preceded by a flag, which tells the client or server how to process the following data. The “hello_l” flag tells a server that a login process is starting. The “response_1” flag is a response from the server; in the TFHVA scheme, this is followed by the tokenized data needed to create the client key and local server key. In the regular login system, this simply tells the client the server is ready to login. The “response_c” flag carries respective authentication data. Lastly, “logic_success” simply notes that the authentication process has worked. The “|” is simply used to separate distinct data sent within the same packet between the client and server or vice-versa; it is tokenized once received to sort out the data which it separates.

The above Figure 14 displays the data intercepted between the client and the server utilizing the scheme outlined in the paper. In order to prove the consistent, dynamic security between sessions, two different sessions were initialized for login. As one can see, not only is the sensitive data fully encrypted between the two despite the lack of SSL protection, but the data is also different with each login, ensuring that data could not be reused in a replay attack, besides also thwarting an attempt to sniff sensitive data. There is also no hint as how to proceed with cracking the received hashes because of the varied pads used in their encryption.

Interceptions for the traditional hash methodology can be seen in Figure 15 and Figure 16. Two different iterations were employed; one which did the password hash work on the client-side (Figure 15) and one which did so on the server-side (Figure 16). Figure 16 provided slightly more security than Figure 15, but is still susceptible to additional attacks. Not only is the hash value consistent between versions, allowing for a replay attack, but the hash can be cracked by running it through either existent hash databases, or through brute-forcing. While the TFHVA scheme shown in Figure 8 can theoretically be brute-forced as well, the dynamic element alongside the pad creates an exponentially increased difficulty for attackers since the password is not static.

## 7. Conclusions

We introduced seven factors which we attempted to adhere to within the proposed TFHVA scheme. The scheme is primarily based on two concepts, hybrid encryption and hash chaining, and works in a similar methodology to other forms of secure cross-communication, such as aforementioned SSL and TLS but in a decentralized manner, meaning certificates are not needed. Hash chaining is employed in a subtle methodology; instead of mutual iterative chaining, which would theoretically be susceptible to heuristic analysis, the scheme deploys an algorithmic version. Hybrid encryption is deployed through two different standards; symmetric-key encryption is primarily used, but asymmetric encryption is used in generating keys. The scheme works through a multi-factor verification based on segmented mutual data between nodes. A mutual key, similar to a password, is associated with a client in a server data. However, the key is kept encrypted with a rotating randomized one-time-pad between sessions. The mutual key—the encrypted and unencrypted versions—exist in a cyclical algorithm from which derivative key halves, the IoT device key and server key, can be recreated using the algorithmic functionality of the mutually shared algorithmic hash chains. The end goal of the cycle is to recreate the data from which the halves are derived, which can then be used to recreate the one-time-padded key.

We evaluated the scheme in context of the following factors: (i) our scheme does not inflate user responsibilities or add complexities, as they are only responsible for reproducing their user identifier, similar to current-day password-practices. (ii) This scheme does not append or change architecture or structural aspects of current client–server communication model. (iii) This scheme does not sacrifice processing speed as it provides a more secure communications. (iv) The proposed scheme can be easily and flexibly integrated with other schemes to combat other cyber-attacks, as explained through how biometric or graphical interfaces can be paired together to further combat brute-forcing. (v) This scheme out-performed other prototypes [38] and [42], as well as other contemporary methods which were studied, as well as provided resilience to the focused fields of eavesdropping, impersonation, and interception mechanically. (vi) The scheme did not create any additional vulnerabilities or weaknesses to other attacks which the scheme was not explicitly focused on addressing, such as brute-forcing, and (vii) it provides a strong resilience to cyber-attacks (as in Section 5, as well as the proof-of-concept implementation within Section 6) without any additional middlemen, proprietary or distinctive software, or complex algorithmic functionalities. We have created and shown a proof of concept as a testable system that does not burden the user or require any alterations to any client–server architectures. Where this system does show weakness, augmenting methodologies can be used to circumvent weaknesses; for example, the usage of 2FA/MFA, and flexible front-facing interfaces for authentication. Furthermore, this form of authentication allows for secure session management and an increased security in stored data on server-side databases.

## Figures and Tables

**Table 1:** Notations.

User Identifier	UI	One Time Pad	O_TP_
Hashed User Identifier	UI_H_	AND	∧
Reconstructed User Identifier	_R_UI	Server Key	S_k_
Hashed reconstructed user identifier	_R_UI_H_	Client key	C_k_
Transaction store	TS	Reconstructed client key	_R_C_k_
Hashed transaction store	TS_H_	Identifier	Idn
XOR	⊻	Reconstructed identifier	Idn_R_
Padded client key	_P_C_k_	Reconstructed server key (client recreation)	_R_S_k_
Derivation function	F_1_	Inverse derivation function	F_2_
Random generated	RND	Encrypted hash identifier	EHI
Unencrypted payload	P	Session initializer	SI
Constructed server key (validation on server)	_C_S_K_		

**Table 2:** Time complexities.

Protocol	Time Complexity
TFHVA	O(n × (m + l))
Password Hash Comparison (No Salt)	O(n × m)
Password Hash Comparison (with Salt)	O(n × m)
Two Factor Password Security	O(n_1_ × n_2_ × n_3_ × m)
Attempt Based Password [42]	O(n × m)

**Table 3:** Data stored and transferred within each protocol, as well as data user is responsible for.

Protocol	Data Transferred	Stored Data (Server)	User Responsibilities
TFHVA	From server; hash of transaction store. From client; padded client key.	Server key, transaction store	User identifier
Simple Login System (Password Hash Comparison)	From client; Plaintext password or hash of password.	Hash of password	User password
Two Factor Password Security (with Image) [38]	Password, image	Secret key, key derivative, password derivative	User password, user image
Two Factor Password Security (with Text) [38]	Password, password key	Secret key, key derivative, password derivative	User password, user token
Attempt Based Password [42]	Password(s)/password attempts - cleartext or hashed	Password(s), mapped attempt(s)	User password, attempt number, nuances in attempt passwords

**Table 4:** Protocol solutions.

Protocol	Strengths	Vulnerabilities/Weaknesses
TFHVA	Resilience to man-in-the-middle attacks, replay attacks. Flexible user identifier generator resists against brute forcing. Sensitive data is never transferred or directly stored.	Only as strong as the method of generating the original user identifier. Vulnerabilities solely rely on reproduction of user identifier rather than interception or transactional risks.
Simple Login System (Password Hash Comparison)	Speed, small data transfer, minimal user responsibility	Man-in-the-middle attack, replay attack, brute forcing/dictionary attack, key logging, insecure password storage, weak/cracked user password
Two Factor Password Security (with Image) [38]	Resistance to unauthorized login attempts, resistance to key-logger, online/offline implementation, password not stored	Increased complexity for users. Man-in-the-middle attack, replay attack. Insecure server data storage.
Two Factor Password Security (with Text) [38]	Resistance to unauthorized login attempts, resistance to key-logger, online/offline implementation, password not stored	Increased complexity for users. Man-in-the-middle attack, replay attack. Insecure server data storage.
Attempt Based Password [42]	High resistance to brute-forcing	Man-in-the-middle attack, replay attack, key logging, insecure password storage, weak/cracked user password

**Table 5:** Communication times; times averaged across 15 attempts for each method.

Protocol	Remote Authentication Time (Average)	Local Authentication Time (Average)	Number of Cryptographic Operations During Authentication
TFHVA	0.0042 s	0.0033 s	4 operations (contains additional-non-cryptographic operations as well)
Simple Login System (Password Hash Comparison)	0.0037 s	0.0024 s	1 operation (hash generation)
Two Factor Password Security (with Image) [38]	1.6744 s	0.9371 s	2 operations
Two Factor Password Security (with Text) [38]	0.0044 s	0.0031 s	2 operations
Attempt Based Password [42]	0.0042 s	0.0021 s	N, where n is equal to the amount of required attempts
